# Revisiting the Role of Vitamins and Minerals in Alzheimer’s Disease

**DOI:** 10.3390/antiox12020415

**Published:** 2023-02-08

**Authors:** Harsh Shah, Fereshteh Dehghani, Marjan Ramezan, Ritchel B. Gannaban, Zobayda Farzana Haque, Fatemeh Rahimi, Soheil Abbasi, Andrew C. Shin

**Affiliations:** 1 Neurobiology of Nutrition Laboratory, Department of Nutritional Sciences, College of Human Sciences, Texas Tech University, Lubbock, TX 79409, USA; 2 Department of Nutritional Sciences, School of Nutrition and Food Sciences, Kermanshah University of Medical Sciences, Kermanshah 67158-47141, Iran

**Keywords:** essential micronutrients, antioxidants, anti-inflammatory, amyloid, Tau, cognitive function

## Abstract

Alzheimer’s disease (AD) is the most common type of dementia that affects millions of individuals worldwide. It is an irreversible neurodegenerative disorder that is characterized by memory loss, impaired learning and thinking, and difficulty in performing regular daily activities. Despite nearly two decades of collective efforts to develop novel medications that can prevent or halt the disease progression, we remain faced with only a few options with limited effectiveness. There has been a recent growth of interest in the role of nutrition in brain health as we begin to gain a better understanding of what and how nutrients affect hormonal and neural actions that not only can lead to typical cardiovascular or metabolic diseases but also an array of neurological and psychiatric disorders. Vitamins and minerals, also known as micronutrients, are elements that are indispensable for functions including nutrient metabolism, immune surveillance, cell development, neurotransmission, and antioxidant and anti-inflammatory properties. In this review, we provide an overview on some of the most common vitamins and minerals and discuss what current studies have revealed on the link between these essential micronutrients and cognitive performance or AD.

## 1. Introduction

In the past few decades, numerous scientific technologies and discoveries have led to the development of various lifestyle, pharmacological, and surgical strategies to effectively treat many chronic disorders such as cardiovascular, respiratory, and metabolic diseases. The medical advancement with successful therapeutics has clearly helped enhance our longevity with the increase in global life expectancy from 62.8 years in 1980 to 72.7 years in 2020 [1]. However, it also inevitably increased in parallel the number of older individuals with dementia, an umbrella term used to describe abnormal age-related conditions that substantially impact cognitive functions. Alzheimer’s disease (AD) is the most common type of dementia that affects over 6 million Americans in the US alone and 50 million individuals worldwide. It is an irreversible, debilitating neurodegenerative disease that is characterized by memory loss, impaired learning and thinking, and difficulty in performing regular daily activities [2]. Currently, there are no cures or effective medications to prevent or treat AD, which translates into USD 321 billion in healthcare costs in the US and over USD 1 trillion in the world [2] that places a significant financial and psychological burden on both patients as well as their family members or caregivers. Essentially all clinical trials in the last two decades (except for the recent controversial Aducanumab and Lecanemab) that targeted key pathological hallmarks of AD including amyloid plaques, Tau protein, and neuroinflammation ended without success [3], indicating that we still lack a complete understanding of the pathophysiology of this devasting disease.

AD is without a doubt a complex, heterogeneous disease with many risk factors, and the link between nutrition and AD has been relatively recently highlighted as we gained more understanding of the critical role of nutrition on brain health [4]. For instance, our brain utilizes approximately 20% of glucose during a resting state every day, and a sufficient amount of various amino acids and lipids are necessary to execute optimal enzymatic reactions for a myriad of cellular/metabolic functions and for myelin maintenance for axonal transport and neurotransmission [5,6]. In addition, the type and quality of nutrients an individual consumes are another important determinant that governs brain health. The Mediterranean diet that puts emphasis on plant-based foods and healthy fats has recently been shown to significantly reduce brain atrophy in male adults, in particular the hippocampus which is a brain region mainly responsible for learning and memory and one of the first areas affected by AD [7]. The DASH diet is another healthy diet choice that has been shown to be associated with superior global cognition and verbal memory [8], as well as a slower rate of decline in global cognition and episodic/semantic memory [9]. Therefore, it comes as no surprise that nutrients and their metabolic consequences play a pivotal role in maintaining proper brain functionality. This notion is further supported by studies revealing that metabolic disorders (ex. obesity, type 2 diabetes) that exhibit impaired nutrient handling and/or metabolic pathways serve as strong risk factors for AD [10].

While eating balanced nutrients is therefore essential for proper CNS neurotransmission and plasticity, this would not be accomplished if the nutrients are not transported to specific tissues/cells and metabolized appropriately through the dedicated enzymes and signaling pathways. Vitamins and minerals sit at center stage in this process due to their indispensable role in catalyzing nutrient utilization and serving as a defense calvary in response to cellular injury and dyshomeostasis brought about by the oxidized byproducts [11]. Vitamin B is a prime example in which actions range from carbohydrate metabolism and amino acid breakdown to nutrient transport throughout the body. Likewise, being a redox catalyst, vitamin C works as an excellent scavenger of free radicals that are generated during cellular metabolic processes. Minerals or trace elements that are found in dietary sources are also known to play a vital role in facilitating metabolic reactions and neurotransmission, as well as in alleviating oxidative stress [12].

Current studies provide evidence that circulating levels of certain vitamins and minerals are markedly altered in people with AD compared to those in healthy individuals, raising a possibility that a gradual depletion/excess of these essential micronutrients may act as a potential contributing factor in AD pathogenesis. This notion is in keeping with many studies that have suggested that neural oxidative imbalance and inflammation play a central role in the pathophysiology of AD, with supporting glial cells in the brain put into an overdrive mode [13]. Along with the damaged blood–brain barrier (BBB), the overproduction of free radicals and pro-inflammatory mediators by hyperactivated astrocytes and microglia promote the formation of insoluble amyloid peptides and neuronal death. Hence, it is conceivable that micronutrients with antioxidant, anti-inflammatory, and/or anti-apoptotic properties may be able to at least partly mitigate AD development (Table 1). This review summarizes the role of some of the widely known vitamins and trace elements and discusses our current knowledge of their correlational and/or causal links with cognitive functions and AD development based on the existing human, animal, and cell studies.

**Table 1:** Summary on the role of vitamins in AD, cognition, amyloid β, and Tau pathology.

Intervention	Vitamins	Human Studies	Animal Studies
Dietary intake/ supplementation/ higher circulating levels	A	Associated with ↑ cognition [14]	↓ Aβ, ↓ pTau, ↑ cognition [15,16]
	C	Positive [17,18], weak or no [19,20,21] association with AD or cognition	↓ Aβ, ↓ pTau [22]
	D	Positive association [23], ↓ Aβ, ↑ cognition [24,25]	↑ Cognition [26]
	E	Positive [27,28] or no association [29,30,31,32] with cognition or AD, delays cognitive decline [33,34,35]	↑ Cognition [36,37,38]
	K	↑ Cognition [39,40,41,42,43]	-
	B1	↓ Cognition [44]	↓ Aβ [45]
	B3	Associated with ↑ cognition [46]	↑ Cognition, ↓ pTau [47,48]
	B6	-	↓ Aβ, ↓ pTau, ↑ cognition [49] ^&^
	B12	↑ Cognition [50] *	-
Deficiency/ restriction/ lower circulating levels	A	Associated with AD [51,52,53,54,55,56]	↑ Aβ, ↑ pTau, ↓ cognition [55,57,58,59]
	C	Associated with AD [60]	↓ Dopamine [61]
	D	Associated [62,63,64] or no association [65,66,67,68] with cognitive decline	↑ Aβ, ↑ pTau, ↓ cognition [69]
	E	Associated with AD [54,70,71,72]	↑ Aβ, ↓ cognition [73,74]
	K	-	-
	B1	Associated with AD [75]	↑ Aβ, ↓ cognition [76,77]
	B3	-	↑ Aβ [78]
	B6	-	↑ Aβ, ↑ pTau, ↓ cognition [79,80,81] ^&^
	B12	Positive association [82,83,84,85]	↑ Aβ, ↓ cognition [86,87] *

* Along with folate, ^&^ along with folate and B12, ↑ = increase Aβ/pTau or improved cognition, ↓ = decrease Aβ/pTau/dopamine or impaired cognition, Aβ—amyloid beta, pTau—phosphorylated Tau.

## 2. The Association between Vitamins and AD

### 2.1. Vitamin A

Synthesized and stored mainly in the liver, vitamin A (retinol) is a fat-soluble vitamin that has a crucial role in the development of the CNS by promoting axonal growth, neural differentiation, and maintenance [88]. Current evidence suggests a positive association between low circulating vitamin A levels and AD [51,52,53,54]. More recent studies extend this finding by demonstrating that vitamin A is also reduced in elderly patients with mild and moderate cognitive impairment (MCI) [14,55,56,89]. Interestingly, others have found that not serum retinol levels, but its precursor carotenoids are significantly associated with cognitive performance in centenarians (i.e., individuals with age ≥ 98) [90]. Another study [91] failed to reveal any relationship between vitamin A or its precursors and cognitive deficit in an elderly Korean cohort, although the interpretation is difficult since the participants had a mean score over 25 based on the Mini-Mental State Examination (MMSE) which is considered cognitively healthy.

Using animal models of AD, investigators have observed a causal link between vitamin A and AD progression. A regular chow diet deplete of vitamin A in an APP/PS1 mouse model resulted in increased Aβ peptide production and Tau phosphorylation in the brain [55,57,58,59], consequently leading to learning and memory deficits as evidenced by the increased escape latency in Morris Water Maze [55,57]. On the other hand, vitamin A supplementation has been to be effective in lowering cognitive decline and AD pathology. Six months of vitamin A-rich diet in 3xTg mouse model of AD resulted in enhanced RXR expression in the hippocampus, lowered Aβ and phosphorylation of Tau in the hippocampus, and preserved spatial memory [15]. Five-month-old APP/PS1 transgenic mice treated with All-trans retinoic acid (ATRA) intraperitoneally for eight weeks displayed a marked decrease in Aβ deposition, Tau phosphorylation and cdk5 activity, microglial and astrocyte activation, as well as a reversal of their learning and memory deficits [16]. ATRA also showed promising results in reducing memory deficits and rescuing key biochemical and histopathological changes such as oxidative stress and acetylcholine neurotransmitter synthesis/degradation in streptozotocin-induced dementia [92], suggesting that ATRA is a potential anti-cholinesterase and antioxidant agent for treating people with or susceptible to cognitive impairment.

Circulating retinol stays bound to retinol-binding protein 4 (RBP4) that can be taken up by RBP receptor on the cell membrane (STRA6) [93]. A high expression of STRA6 at the blood–brain barrier (BBB) and circumventricular organs such as choroid plexus allows easy access of retinol to the brain [94,95]. Once metabolized to retinoic acid (RA) inside the cell, it is translocated into the nucleus and binds to a heterodimer complex comprising retinoic acid receptor (RAR) and retinoic X receptor (RXR) to affect gene transcription. While the underlying mechanisms are not clear, the downregulation of key enzymes in the brain such as A disintegrin and metalloproteinase 10 (ADAM 10), insulin-degrading enzyme (IDE), and brain-derived neurotrophic factor (BDNF) that regulate Aβ production and cleavage indicates an amyloid-promoting action in the absence of vitamin A [55,57,58,59]. In support of these findings, reinstating normal vitamin A levels was able to restore the protein abundance of these enzymes that most likely ameliorated the cognitive dysfunction in mice [55,58,59]. Relevant to this, Biyong and colleagues have also shown that RXR undergoes protein modification in the inferior parietal cortex that coincides with the severity of cognitive decline and accumulation of senile plaques in the cortex, suggesting that vitamin A signaling itself may be impaired [15]. Another alternative mechanism may involve gut microbiota. Feeding APP/PS1 mice a vitamin A-deficient diet for 45 weeks dramatically reduced retinol concentrations in the liver and serum and worsened their memory deficits [57]. Importantly, this was associated with decreased expression of BDNF in the cortex and hippocampus and alterations in gut microbial species, in particular, a significant reduction of *Lactobacillus*. Considering that *Lactobacillus* is capable of enhancing intestinal mucosal barrier function [96] and inducing BDNF in the brain [97], these results suggest that vitamin A may regulate the gut–brain axis by promoting healthy gut bacterial species to maintain properly functioning CNS.

In vitro studies have provided some insights into the cognitive improvement following vitamin A supplementation. ATRA treatment to cortical pyramidal neurons for 6–10 h induced ultrastructural remodeling of the calcium-storing spine apparatus organelle in dendritic spines leading to the increased size of dendritic spines and improved synapse plasticity [98]. Vitamin A is also able to directly inhibit Aβ formation and oligomerization and destabilize previously formed Aβ fibrils [99,100]. In line with these findings, Alam and colleagues [101] demonstrated that vitamin A can modify Aβ-42 toward non-toxic aggregates through hydrophobic interactions and hydrogen bonding. There are, however, other studies that indicate neurotoxicity driven by high levels of vitamin A [102]. Collectively, current data suggest that vitamin A has a promising therapeutic potential in treating AD primarily via its anti-amyloid/Tau, antioxidant, and pro-cholinergic effects if given at a safe dose.

### 2.2. Vitamin C

Vitamin C is a water-soluble vitamin that has a wide range of essential roles from the formation of blood vessels and development of cartilage and collagen to induction of iron absorption and regulation of blood pressure, although it is probably best known for its antioxidant and anti-inflammatory effects [103,104]. Emerging studies have begun to elucidate its protective effects in neurodegeneration. A recent study by De Nuccio and colleagues [105] reported that treatment with vitamin C by oral gavage for just 10 days in an MPTP-induced mouse model of Parkinson’s disease is sufficient to substantially decrease dopaminergic neuronal loss and reduce NLRP3 inflammasome activation and pro-inflammatory mediators such as IL-6, TNF-α, iNOS, and TLR4. These pro-neuronal effects were likely responsible for partially alleviating gait and spontaneous locomotor deficits. Consistent with these findings, a high dose of intraperitoneal vitamin C injection in rats with sepsis-induced cognitive impairment was found to attenuate BBB disruption, oxidative stress, and neuroinflammation in the hippocampus while enhancing anti-inflammatory molecules such as superoxide dismutase and IL-10 [106]. In addition to its excellent free radical-scavenging properties, other studies have focused its role on neurotransmission. Daily oral gavage of vitamin C with few other antioxidants including epigallocatechin-3-gallate (EGCG), vitamin E, and selenium (Se) for 5 weeks in aluminum chloride-induced AD rats was able to markedly lower AD-related brain pathology and improve monoamine neurotransmitter levels (DA, NE, 5-HT) in the brain [22]. Along with vitamin C, the anti-inflammatory properties of EGCG and the anti-oxidative role of vitamin E and Se also may have contributed to improving synaptic and cognitive functions as suggested by several studies [107,108,109,110]. These beneficial results are also supported by a loss-of-function study [61] that demonstrated that APP/PS1 mice display reduced dopamine release in the nucleus accumbens, a central hub for motivation and reward, and those mice with impaired vitamin C synthesis by genetic deletion of gulonolactone oxidase have a further reduction of dopamine and their metabolites including DOPAC, 3-MT, and HVA in the ventral striatum.

In contrast to animal studies, the association between vitamin C and cognitive health and the neuroprotective role of vitamin C in humans has yet to reach a consensus. In a small cross-sectional study with a Japanese cohort, vitamin C in both serum and lymphocytes showed a rather weak correlation with the Mini-Mental State Examination (MMSE) scores [19]. Nurses’ Health Study that longitudinally monitored vitamin C and carotenoid intake in nurse participants failed to find the benefit of antioxidant intake on cognitive health [20]. Moreover, a large randomized, controlled trial demonstrated negligible effects of vitamin C supplementation on cognitive functions in the elderly at a dose of 500 mg/day for 6 years or longer [21]. However, a recent cross-sectional study conducted in Cuba reported significantly lower serum vitamin C levels in patients with AD compared to those in healthy age-matched controls [60]. These conflicting results may partly stem from potential sex differences and susceptibility genes. A population-based prospective study in Japan found that high circulating levels of vitamin C are significantly correlated with a decreased risk of apolipoprotein E4 (ApoE4)-associated cognitive decline in women but not men [17]. Liu and colleagues in their Mendelian randomization study revealed a causal association between plasma levels of vitamin C and the risk of AD and its proxy phenotype [18]. Clearly, more controlled clinical studies that target the effects of vitamin C on brain pathology and cognition accounting for sex and ethnicity variables are warranted.

### 2.3. Vitamin E

Vitamin E is a fat-soluble vitamin that has eight analogs including tocopherol (α, β, γ, and δ) and tocotrienols (α, β, γ, and δ) [111]. Being the most abundant form of vitamin E, α-tocopherol is found in dietary sources such as vegetable oils, nuts, and seeds. Vitamin E has been shown to exert antioxidant and anti-inflammatory properties that may potentially provide protective effects against neurodegeneration [111,112]. Patients with AD have lower circulating levels of vitamin E in cerebrospinal fluid (CSF) [70] and serum [71]. In support of this, three meta-analysis studies showed significantly lower concentrations of circulating vitamin E in AD patients or those with age-related cognitive decline compared to the healthy control group [54,72,113]. On the other hand, the relationship between vitamin E and the risk of AD is inconsistent. Mangialasche and others [27] reported that high plasma levels of vitamin E are associated with reduced risk of AD in advanced age. Similarly, a higher intake of vitamin E and α-tocopherol equivalents was found to be associated with reduced AD incidence [28], although other studies failed to observe any significant link between the supplementation of vitamin E and the risk of dementia or AD [29,30,31]. Randomized Mendelian analysis using a large-scale genome-wide association study (GWAS) dataset also indicated no significant association between vitamin E and AD risk based on the inverse-variance and weighted median analyses [32]. It is interesting, however, that AD patients with the use of vitamin E (2000 IU/d) for 15 years had a significantly longer survival rate compared to the control group [114], suggesting a possible ability of vitamin E to increase resilience rather than prevent cognitive decline.

Direct protective, anti-AD effects of vitamin E have been reported in several controlled clinical studies. In the TEAM-AD VA cooperative randomized trial, α-tocopherol supplementation at a dose of 2000 IU/d for over two years was shown to be significantly effective compared to placebo at delaying cognitive impairment among mild to moderate AD patients [33]. A randomized, double-blind, placebo-controlled multicenter trial demonstrated that supplementing subjects with moderate AD with α-tocopherol (2000 IU/d) for two years significantly delayed disease progression as assessed by the ability to perform basic activities of daily living and CDR questionnaires [34]. Further supporting the neuroprotective role of vitamin E, Lloret and colleagues [35] demonstrated that following vitamin E supplementation at a dose of 800 IU/d for six months, AD patients that responded to vitamin E by lowering oxidized glutathione levels in blood (i.e., less oxidative stress) were able to maintain the cognitive status. The timing of vitamin E supplementation with respect to cognitive deterioration seems to be important since supplementation at 400 IU/d for over 5 years did not result in any preventive effect on the incidence of AD or dementia in the PREADVISE study. These findings suggest that vitamin E may be more effective at serving as an AD treatment rather than an AD preventive strategy. On the other hand, a meta-analysis study [115] that included 19 clinical trials showed an increased risk of all-cause mortality due to a high dose of vitamin E supplementation (>400 IU/day). This outcome may be related to the pro-oxidant effects of high doses of vitamin E [116,117,118]. Hypervitaminosis E has been shown to be associated with intracerebral hemorrhages as well [119]. Although we do not yet have any evidence showing the negative effects of high vitamin E on Alzheimer’s disease, caution is warranted for future clinical studies.

The essential role of vitamin E in mitigating AD-related pathology is strongly supported in rodent studies. APPswe mice that are missing tocopherol transfer protein (APPswe:Ttpa^−/−^ double transgenic) are depleted of vitamin E, leading to exacerbated lipid peroxidation and Aβ accumulation in the brain compared to APPswe control mice due to decreased Aβ clearance [73]. Importantly, these mice developed earlier and more severe cognitive dysfunction in Morris Water Maze and contextual fear conditioning compared to a single mutant APPswe mouse, which was completely normalized in mice treated with vitamin E [74]. Similar cognition-preserving effects of vitamin E were also demonstrated in APP/PS1 mice [36,37]. Hippocampal injection of Aβ (1–40) in rats impaired novel object recognition memory and increased oxidative stress, but 14-day treatment with vitamin E helped reduce oxidative stress by decreasing malondialdehyde (MDA) and increasing superoxide dismutase (SOD) that was associated with less neuronal loss and improved memory [38]. The capacity of vitamin E as an antioxidant to reverse AD pathology was confirmed in an in vitro study in which Aβ (1–42)-treated neuronal cultures exhibit increased ROS, protein oxidation, and cell death which could be ameliorated by treatment with vitamin E [120]. In agreement with these results, other studies have also demonstrated the ability of vitamin E to enhance cholinergic neurotransmission in the brain, lower plasma pro-inflammatory mediators, and induce ROS-scavenging activity in AD rats [121,122]. These studies used supplementation of pycnogenol (PYC), an extract of French maritime pine bark, or acetyl-L-carnitine (ALC) and α-lipoic acid (ALA) along with vitamin E. It is likely that the antioxidant effects of PYC [123] and ALC [124] partly contributed to the beneficial action of vitamin E. The effects of vitamin E can further be enhanced by supplementing ALA as ALA can further increase vitamin E’s bioavailability by regenerating vitamin C and glutathione [125]. Further, ALA supplementation in addition to acetylcholinesterase inhibitor (AChEI) in patients with AD and related dementia slowed down further cognitive decline [126]. This result may be attributed to its ability to increase acetylcholine production and reduce oxidative stress [127].

### 2.4. Vitamin K

Vitamin K is another fat-soluble vitamin that is involved in antiapoptotic and anti-inflammatory pathways along with neural development and survival [128]. The two natural dietary forms of vitamin K are phylloquinone (vitamin K1) and menaquinones (vitamin K2) [129]. Dark green leafy vegetables, fermented foods, cheeses, eggs, and meats are the main dietary sources of vitamin K [130]. It is known to regulate key enzymes involved in sphingolipid metabolism, and alteration in the expression of sphingolipids is associated with neuroinflammation and neurodegeneration [128]. Vitamin K status has been linked with general cognitive performance. Higher serum levels of phylloquinone, an indicator of vitamin K status, were associated with better performance in the verbal episodic memory test during aging [39]. In The ELDERMET cohort, elderly individuals with higher levels of serum phylloquinone had a significantly better cognitive function [40]. Additionally, higher postmortem brain concentration of Menaquinone-4 (MK4) was associated with better cognitive function, lower risk of dementia and AD global pathology scores, and fewer neuronal neurofibrillary tangles [131]. Of note, a longitudinal study with six year follow-up showed that dephosphorylated, uncarboxylated matrix Gla protein (dp-ucMGP), another surrogate marker of vitamin K status, was not significantly associated with cognitive decline in middle-aged adults [132], indicating vitamin K-related protein specificity coupled to cognitive functions.

Elevated plasma/serum levels of vitamin K in aged, cognitively intact individuals may be attributed to greater dietary consumption of vitamin K. Indeed, its daily intake was significantly lower in AD patients compared to the control group [133]. Furthermore, The CLIP (Cognition and LIPophilic vitamins) study was able to demonstrate that higher dietary intake of phylloquinone is associated with better cognition as evidenced by higher MMSE scores and lower FBRS scores in older adults [41]. Soutif-Veillon and colleagues [134] reported that greater intake of vitamin K is significantly associated with fewer and fewer subjective memory complaints. The NHANES study revealed similar results with greater vitamin K intake from vegetables [135]. Interestingly, specific isoforms of menaquinones that contain longer chains were found to be positively correlated with cognitive performance based on the subset data from the ELDERMET study [42]. A potentially causal relationship between vitamin K and AD or cognitive decline was examined in geriatric participants that had used warfarin, acenocoumarol, or fluindione—vitamin K antagonists (VKA)—over 24 months. Compared to non-VKA users, these subjects presented lower scores on the Frontal Assessment Battery which is indicative of worse executive dysfunction [43].

A body of in vitro studies shed light on the possible mechanisms by which vitamin K may improve or maintain cognitive health. Treating human neuroblastoma MSN cells with menadione (i.e., vitamin K3) induced dose-dependent thiol oxidation and dephosphorylation of Tau [136]. In keeping with its AD-counteracting effects, vitamin K2 supplementation in astroglioma C6 cells transfected with C-terminal fragment of APP was able to dose-dependently decrease cell death and ROS formation induced by Aβ peptides [137]. The protective mechanism is likely through regulation of phosphatidylinositol 3-kinase (PI3K) signaling pathway and inhibition of caspase-3-mediated apoptosis. Furthermore, pre-treating pheochromocytoma PC12 cells with vitamin K2 significantly reduced Aβ (1–42), ROS, H_2_O_2_ cytotoxicity, apoptosis, and inactivated p38 MAP kinase pathway [138]. A rescue of Aβ-induced neurotoxicity by K2 pre-treatment was recapitulated in the transgenic Drosophila model of AD most probably through activation of autophagic pathways [139]. Other isoforms of vitamin K have also demonstrated anti-inflammatory and anti-amyloid aggregating effects in various cell lines including HEK293, erythrocytes, and neuroblastoma SH-SY5Ycells [140,141,142].

### 2.5. Vitamin B12

Vitamin B12 (B12) is an essential water-soluble organic compound that is known to be critical for DNA synthesis, methylation, and cellular metabolism. Major complications due to B12 deficiency are megaloblastic anemia and neurological problems [143]. Unlike deficiency, inadequate B12 or a subclinical deficiency is very common in elderly populations [144], and a number of observational studies have shown an association between B12 deficiency with AD [82]. A retrospective study of patients with familial AD showed significantly low serum B12 levels compared to their unaffected family members [83]. A similar correlation is also evident in older individuals with sporadic AD, the most common form of the disease. A population-based longitudinal study in Sweden demonstrated that cognitively intact subjects (≥75 years old) with lower B12 at baseline exhibit twice higher risks of developing AD compared to people with normal levels of B12 within 3 years [84]. In agreement with the inverse relationship, another study with a 10 year follow-up found that the subclinical B12 deficiency precedes cognitive decline in elderly populations [85]. B12 deficiency in AD is also associated with elevated plasma levels of TNFα and IL-6 [145,146], two main pro-inflammatory mediators that have been shown to increase hyperphosphorylation of Tau and induce Aβ synthesis [147,148,149].

Homocysteine is a non-proteinogenic amino acid that is converted from methionine by B12 during folate metabolism, thus allowing us to use its circulating amount as a surrogate marker of B12 levels. Clarke and colleagues [150] have found that patients with AD have significantly higher serum homocysteine levels compared to healthy control subjects which are accompanied by markedly lower serum B12 and folate, and that was associated with cortical atrophy in the temporal lobe [150]. A retrospective analysis of plasma samples from the Framingham Study showed increased homocysteine levels as a strong independent risk factor for the development of AD [151]. Likewise, in a study conducted by Annerbo and others [152], people with high homocysteine levels have a two-fold higher risk of developing AD compared to those with low homocysteine.

Studies that involved B12 supplementation have shown beneficial effects on cognition and inflammatory status. A randomized, single-blinded placebo trial in patients with clinically diagnosed AD showed improvement in cognition upon six months of folate and B12 supplementation [50], and this was associated with decreased serum homocysteine and TNF-α. Moreover, supplementation of B12 in combination with an antipsychotic therapy significantly lowered psychotic symptoms and promoted anti-inflammatory cytokines while reducing pro-inflammatory cytokines [153], pointing to an immune-modulatory role of B12.

The positive effects of B12 are further supported In animal studies. APPswe mouse model of AD that is fed with a diet deficient of folate, B12, and B6 displayed higher Aβ1–40 levels in the hippocampus and Aβ1–42 levels in the cortex, indicative of accelerated amyloidogenic phenotype that is a key pathological feature of AD [86]. Similarly, memory impairment induced by Aβ peptide infusion directly into the hippocampus was exacerbated in rats that were placed on a folate and B12-deficient diet [87]. B12 pre-treatment in scopolamine-induced AD rats significantly reduced inflammatory and apoptotic markers and preserved protein expression of synaptic proteins, suggesting a neuroprotective role of B12 [154]. Since inadequate B12 leads to high plasma homocysteine levels that can induce oxidative stress [155,156,157], it is likely that B12 has antioxidant and anti-inflammatory properties via both direct and indirect pathways.

Altogether, these studies indicate that high homocysteine and low B12 may potentially contribute to the disease progression, although the isolated effect of B12 on AD-related cognitive deficit is somewhat questionable. In a longitudinal study in 2017, the dietary intake of vitamin B and circulating B12, folate, and homocysteine levels in AD patients were measured in a 13-month period. Their plasma homocysteine levels significantly increased along with cognitive decline without any changes in B12 intake or its plasma levels, suggesting a contribution of unknown non-dietary factors [158]. It is possible that the metabolic function of B12 is already impaired once an individual is diagnosed with AD which may partly explain hyperhomocysteinemia in AD with further cognitive decline that is independent of plasma levels or dietary intake of B12.

### 2.6. Vitamin B6

Pyridoxine (vitamin B6) is a water-soluble vitamin mainly found in fruits, vegetables, and grains, and can also be obtained through supplements. The active form of this coenzyme, pyridoxal-5-phosphate (PLP), is responsible for vital functions including the breakdown of nutrients, synthesis of red blood cells, maintaining normal levels of homocysteine, and supporting immune function and the nervous system [159]. To determine if systemic B6 levels have any beneficial implication on cognitive performance in a healthy aging population, Jannusch and colleagues evaluated blood levels of vitamin B6 and cognitive functions, cortical folding, and functional resting-state connectivity in 794 healthy subjects (55 ≤ age ≤ 85) who were recruited from the population-based 1000BRAINS study [160]. The study found a significant positive correlation between blood B6 levels and local cortical folding throughout the brain without any meaningful association with functional connectivity or neuropsychological test scores. Because aging is linked with brain atrophy and the diminution of folds and grooves within the cortical areas, these findings provide new insight into the possible role of B6 on brain structure during the non-demented aging process. Relevant to the effects of vitamin B6 on age-related cognitive decline, a Cochrane review evaluated 14 randomized controlled trials that assessed cognitive functions of non-demented elderly individuals who used B vitamin supplements for at least three months [161]. The study found that compared to a placebo, vitamin supplements (B6 alone or in combination with B12 and folic acid) did not result in any meaningful beneficial effects on cognitive performance, suggesting that the impact of vitamin B6 may be rather minimal in aging people that are cognitively healthy.

An association between vitamin B6 and the integrity of brain structures in AD was studied in both male and female participants (mean age = 70) who are diagnosed with AD [162]. Assessment of white matter lesions (WMLs) by MRI showed an inverse relationship between blood B6 levels and both periventricular and subcortical WML loads, which are usually caused by problems with the brain vasculature. Whether the observed outcome was partly due to malnutrition in AD individuals is not clear, but it suggests that low B6 levels may potentially promote vascular burden in the AD brains. Vitamin B6 maintains low cellular levels of homocysteine by converting it to cysteine. Increased plasma homocysteine is a strong independent risk factor for the development of dementia [163]. It has been speculated that low blood levels of vitamin B6 are related to cognitive decline possibly due to its inability to remove excess homocysteine. Mulder and colleagues [164] investigated if an association between homocysteine metabolism (i.e., homocysteine, folate, B12, B6) and occurrence of WML exists in patients with AD. The inverse relationship between plasma B6 and periventricular WMLs was confirmed, but they failed to observe a similar association between plasma homocysteine levels and WML. It is not clear why homocysteine could not predict the degree of WMLs, but it may be related to inclusion of AD patients that have homocysteine and related parameters mainly within the normal range. On the contrary, a randomized controlled study reported that higher plasma homocysteine levels at baseline are associated with faster gray matter (GM) atrophy in elderly subjects diagnosed with MCI [165]. More importantly, compared to a placebo group, treatment with a high dose of B vitamins consisting of folic acid, B12, and B6 for 24 months was able to slow down the shrinkage of the whole brain volume, and significantly reduce GM atrophy as much as seven-fold in vulnerable areas such as the medial temporal lobe. Considering the beneficial effects of B vitamins being confined to the subjects with high plasma homocysteine levels, these findings suggest that B6 supplementation may at least partly contribute to delaying the brain atrophy possibly through the reduction of homocysteine. Whether the reduction of cerebral atrophy leads to less cognitive deficits was unfortunately not evaluated, but this was addressed in a randomized, double-blind, placebo-controlled trial in Taiwan [166]. A total of 89 mild to moderate AD patients at 50 years and older were enrolled for 26-week study and were given either a placebo or a multivitamin supplement that contains vitamins B6, B12, and folic acid. The vitamin supplementation was indeed associated with a significant decrease in serum homocysteine levels, however no beneficial effects on cognitive performance or activities of daily living (ADLs) were observed between the two groups.

In animal studies, the role of vitamin B6 has been investigated mainly in the context of AD-related pathology and cognitive deficits induced by various stimuli and stressors. Feeding a B6-deficient diet to C57Bl/6J mice for 4 weeks promoted social defeats and cognitive impairment by inducing noradrenergic imbalance in the prefrontal cortex and striatum [79]. Treatment with a B6 restriction diet for 16 weeks aggravated oxidative stress, amyloid deposition and Tau phosphorylation, and neuronal loss in high fat-fed obese mice [80]. Similarly, 5-week-old mice placed in a pyridoxine deplete diet for 8 weeks led to a significant decrease in the neurotransmitters—serotonin (5-HT) and dopamine (DA)—in the hippocampus compared to normal chow-fed controls [81]. In TgCRND8 mouse model of AD that overexpress APP, deprivation of B vitamins (Folate, B6, B12) from their chow diet induced hyperhomocysteinemia, Aβ accumulation, and impaired spatial memory [167]. On the other hand, the addition of vitamin B6 or the rise of its endogenous levels appears to be neuroprotective and sufficient for memory enhancement. Multi-vitamin B supplementation that contains B6, B12, and folate demonstrated lower Tau phosphorylation and improved memory function in the hypoxia-induced neurodegeneration mouse model [49]. Showing an isolated effect of vitamin B6, intraperitoneal injections of pyridoxine (350mg/kg BW) twice a day for 3 weeks in young C57Bl/6J mice improved novel object recognition memory and significantly raised 5-HT concentrations and the protein expression of tyrosine hydroxylase (TH), the rate-limiting enzyme for catecholaminergic neurotransmitter synthesis, in the hippocampus [168]. Consistent with these findings, high plasma and brain PLP (active form of B6) levels achieved by deletion of PLP-degrading enzyme in PDXP-null mice increased GABA levels in the brain by ~20% and markedly enhanced spatial memory and motor performance [169]. The important role of vitamin B6 in brain health has also been demonstrated in animals other than mice. Young gerbils with surgery-induced ischemia displayed neuronal loss, activated microglia and astrocytes, and lipid peroxidation in the hippocampal CA1 region. Interestingly, mice that were fed a pyridoxine-deficient diet for 8 weeks had significantly higher serum homocysteine levels and further worsened the brain pathology, suggesting that vitamin B6 deficiency accelerates neuronal damage and loss most probably by raising homocysteine levels and lipid peroxidation. Mechanistically, the neuroprotective effects of vitamin B6 may be attributed to its ROS-scavenging action through activation of the antioxidant and anti-inflammatory Nrf2/HO-1 pathway [170].

### 2.7. Vitamin B3

Niacin (vitamin B3) is an essential dietary element taken mainly through diet and supplement sources. The two most common forms of niacin are nicotinic acid and nicotinamide which can be synthesized from the amino acid tryptophan. Niacin plays an important role in converting food into cellular energy, building complex lipids including cholesterol, synthesis, and repair of DNA, and has antioxidant properties [171,172]. Although extremely rare in developed countries, niacin deficiency can cause pellagra, a condition that can lead to depression and memory loss. To date, only a few clinical studies have explored the association between niacin intake and cognitive functions and AD. A prospective study conducted between 1993 and 2002 with participants ≥65 years old observed an inverse relationship between AD and dietary intakes of either total niacin (foods and supplements), niacin from foods only, or tryptophan. Higher niacin intake from food sources was also positively associated with lower cognitive decline [46]. A more recent study by Qin and colleagues [173] evaluated whether the intake of B vitamins including niacin in young adulthood is associated with cognitive functional status in midlife. A cohort of black and white women and men with the age range of 18–30 years old from the Coronary Artery Risk Development in Young Adults (CARDIA) study showed that higher intake of B vitamins and niacin throughout the young adulthood is associated with better cognitive performance later in life via a battery of cognitive and psychomotor tests.

A number of rodent studies have explored the potential therapeutic benefits of niacin and its other forms (i.e., nicotinic acid, nicotinamide) on brain health and neurodegenerative diseases including AD. Green and colleagues examined the efficacy of nicotinamide in restoring cognitive functions associated with AD pathology in 3xTg-AD mice [47]. Treatment with nicotinamide in drinking water for 4 months partially reversed hippocampus-dependent spatial memory impairment as assessed by Morris Water Maze. Likewise, the contextual fear memory test showed that nicotinamide can prevent the loss of fear-associated memory retention in these mice. The cognitive improvement by nicotinamide supplementation was associated with a significant reduction of Tau phosphorylation at threonine residue 231 (pTau 231) which is known to induce microtubule depolymerization in neuronal axons and dendrites. A significant decrease in monoubiquitin-conjugated Tau in nicotinamide-treated 3xTg mice compared to vehicle-treated mice is interpreted as rapid degradation of the phosphorylated Tau by nicotinamide. With the nicotinamide-induced increase in acetylated α-tubulin that is critical for microtubule stability, these findings collectively suggest that oral nicotinamide may be developed as a safe and effective treatment for AD and other tauopathies. Others have investigated the beneficial role of nicotinamide adenosine dinucleotide (NAD+), a niacin-derived coenzyme that is central for cellular energy metabolism, mitochondrial health and biogenesis, gene repair, and neuronal stress resistance. NAD+ supplementation in 17-month-old WTs or 3xTg-AD mice with DNA repair deficiency (3xTg-AD/Polβ^+/−^) through nicotinamide riboside for six months completely restored spatial, recognition, and contextual fear memory [48]. In line with the improved cognitive function, NAD+ treatment significantly alleviated neuroinflammation, impaired synaptic plasticity, DNA damage, and hippocampal neuronal loss in these mice.

Microglia, the resident macrophages in the brain, are activated in response to amyloid pathology partly through the niacin receptor (HCAR2) to exert protective effects. AD mice that have defective HCAR2 display exacerbation of amyloid pathology, neuronal loss, less microglia-mediated Aβ phagocytosis, and accelerated onset of cognitive deficits [78]. A recent study by Moutinho and colleagues [78] draws a great interest regarding AD treatment options by demonstrating that supplementing 5xFAD mice that carry inactivated HCAR2 with Niaspan—an FDA-approved formulation of niacin—significantly reduces plaque burden, neuronal dystrophy, and neuronal loss, while restoring working memory. Testing the efficacy of Niaspan in patients at different stages of AD appears as a desirable therapeutic approach since this formulation is shown to be safe for consumption, although the range of doses needed to effectively reach the brain and the potential impact on peripheral macrophages would first need to be determined.

### 2.8. Vitamin B1

Thiamin (vitamin B1) is naturally found in some food sources and is also commonly used as an added micronutrient for food fortification and available as a supplement. Thiamin is involved in maintaining cellular growth and plays a critical role in converting nutrients to energy. Thiamin deficiency (TD) is causally linked to neurological disorders such as Wernicke–Korsakoff syndrome whose one of the primary symptoms is memory loss [174]. Recently, TD has also been suggested to play a role in AD development mainly in animal studies. TD has been shown to alter APP and/or Aβ metabolism, thereby promoting plaque accumulation independent of neuronal loss. This effect of TD and brain pathology was investigated in Tg19959 transgenic mice overexpressing a double mutant form of APP [76]. Deficiency in thiamin aggravated amyloid plaque pathology localized in the cortex, hippocampus, and thalamus of these mice. TD also led to increased levels of Aβ42, β-secretase cleaved C-terminal fragment (β-CTF99), and β-site APP cleaving enzyme 1 (BACE1 or β-secretase 1) protein that was associated with impaired oxidative metabolism and enhanced oxidative stress. Through an in vitro study, Zhang and colleagues investigated how TD affects APP processing in SH-SY5Y neuroblastoma cells that overexpress APP and found that TD promoted maturation of BACE1 and increased β-secretase activity that resulted in elevated levels of Aβ peptides as well as β-CTF [45]. Importantly, thiamin supplementation was able to reverse the TD-induced stimulation of amyloid peptide synthesis. Consistent with these results, TD in C57Bl/6J mice raised Aβ accumulation in the brain that was completely reversed by thiamin supplementation [45]. TD induction by feeding F344/BN rats a thiamin-deficient diet along with injections of thiamin antagonist produced a time-dependent response, with no change up to 9 days but severe pathological lesions in the thalamus, mammillary body, inferior colliculus, and periventricular areas following 13 days of treatment [175].

Reduced blood levels of thiamin diphosphate (TDP) strongly correlate with brain glucose hypometabolism, and this neurodegenerative feature is tightly linked with cognitive impairment and AD progression [176]. In addition to decreased circulating TDP levels, Sang and others [176] observed a significant decline in brain glucose utilization in thiamin-deprived mice compared to those in control mice as evidenced by reduced uptake of 2-[18F]fluoro-2-deoxy-D-glucose in multiple brain regions. The mechanism by which low TDP is causally linked to AD-related cognitive decline was demonstrated in both in vitro and in vivo settings by lower excitatory neurotransmission and impaired hippocampal long-term potentiation (LTP) following thiamin depletion [77]. Interestingly, treatment with benfotiamine, a synthetic form of thiamin, was able to reverse the neuronal defects. Benfotiamine has been also shown to be effective in alleviating hypothalamic dysfunction in STZ-induced neurodegeneration in rats [177]. While these findings clearly point to low thiamin as a potential contributor to AD pathogenesis, others have hinted that it may be a phenomenon that occurs downstream during AD development. Ramamoorthy and colleagues [178] showed that thiamin transporters 1 and 2 (THTR-1 and THTR-2) are significantly less expressed in the prefrontal cortex and hippocampus of AD patients and 5xFAD mice compared to those in the healthy controls. After confirming this in human neuroblastoma SH-SY5Y cells, they further demonstrated that exposure to pro-inflammatory mediators (IL-1β, IL-6, and TNF-α) to mimic local neuroinflammation led to a marked inhibition of thiamin uptake that was sufficient to alter AD-related genes. Thus, these findings suggest that low thiamin can act as the main driver to induce neuronal damage and also serve as a mediator to exacerbate AD-related pathology during the disease progression.

Thiamin diphosphate (TDP) is an important cofactor for glucose metabolism and is significantly reduced in the brain and blood samples of patients with AD [179]. A case–control study comprising AD subjects (n = 90) and age-matched controls (n = 90) revealed significantly lower blood TDP levels in participants with AD, and interestingly, the levels were even lower in female AD patients compared to male AD patients [75]. The authors did not observe any correlation between TDP levels and several metabolic factors such as fasting glucose, triglyceride, and total cholesterol, but suggested that the lower TDP in females than males may at least partly explain the higher prevalence of AD in females. In another study, 45 AD patients clinically diagnosed and 38 age- and gender-matched control subjects without dementia were voluntarily recruited [51]. The same group has further explored and detected significantly higher thiamin diphosphatase and monophosphatase—the enzymes responsible for inactivating TDP—in AD patients compared to those in the control group, suggesting that either altered or uncontrolled covalent modification in thiamin synthesis may play a role in brain glucose metabolism and susceptibility to AD development.

A limited number of human studies report similar neuroprotective effects of thiamin. Administration of benfotiamine for 18 months has been shown to improve MMSE-based cognitive scores in patients with mild to moderate AD that is independent of brain amyloid accumulation [44]. A recent study led by Gibson and others [180] reported that benfotiamine treatment for 12 months successfully delayed cognitive decline in AD patients compared to the placebo group by 43% based on the Alzheimer’s Disease Assessment Scale-Cognitive Subscale (ADAS-Cog), and resisted the disease progression by 77% based on Clinical Dementia Rating (CDR). Altogether, these findings suggest that thiamin or vitamin B1 is essential for proper glucose metabolism, neurotransmission, and inhibition of amyloid formation, thereby offering a promising outlook of thiamin or its derivatives as an effective therapeutic agent to treat AD and other neurological/neurodegenerative disorders that share similar brain pathology.

### 2.9. Vitamin D

Vitamin D is a steroid hormone mainly recognized for its role in promoting bone health through regulating calcium and phosphate homeostasis. In the past decades, however, many studies have shed light on the novel functions of Vitamin D beyond skeletal growth and maintenance including regulation of cell differentiation, reducing the cardiovascular disease risk, controlling the immune system, attenuating oxidative stress and inflammation, and inducing neuroprotective and neurotrophic effects [181]. While a few studies failed to find a relationship between blood levels of vitamin D and cognitive decline [62,63,64], others have reported a clear association between vitamin D levels and AD or the risk of developing dementia. Several prospective longitudinal studies including the Cardiovascular Health Study, Austrian Stroke Prevention Study, and Rotterdam Study have demonstrated that low serum vitamin D concentrations are linked to a higher incidence of all-cause dementia/AD or lower cognitive functions [65,66,67,68]. Participants (~65 years of age) from a Brazilian cross-sectional study that were diagnosed with dementia showed lower serum vitamin D levels. Interestingly, a rise in each unit of serum vitamin D led to a fall in dementia prevalence by 8%, suggesting that vitamin D may be a meaningful disease-modifiable factor [182]. A recent study by Zhao and colleagues [23] examined if the consumption of vitamin D is associated with the risk of dementia. A multi-ethnic cohort from the Washington Heights-Inwood Columbia Aging Project (WHICAP) comprised more than 1750 individuals over 65 years old without dementia at baseline. At a 5.8-year follow-up, 329 subjects were diagnosed with dementia and those with the lowest vitamin D intake had the highest risk of developing dementia, supporting the concept that higher vitamin D consumption, and its enhanced action thereof, may be beneficial for healthy cognitive functions. This is consistent with the findings from a Mendelian randomization study in which single nucleotide polymorphisms that affect the vitamin D metabolic pathway significantly increase AD risk [183]. The reasons for the lack of associations between vitamin D and AD or dementia from some aforementioned studies are not clear, but they may be attributed to a small sample size, short follow-up periods, having unadjusted covariates, or different methods for assessing 25-hydrovitamin D and dementia.

Thus far, results from the animal studies seem to be in line with those in human studies [26,69,184]. APP/PS1 mice that were treated with vitamin D-deficient diet for 13 weeks displayed low serum levels of vitamin D compared to normal diet-fed APP/PS1 or WT mice that were accompanied by higher expression of inflammation markers such as TNF-α, IL-1β, IL-6, greater Aβ aggregates, increased Tau phosphorylation, synaptic dystrophy, and increased neuronal loss in the cortex [69]. These mice also showed an accelerated cognitive impairment as evidenced by increased escape latency in Morris Water Maze. These results suggest that correcting vitamin D deficiency may allow for prevention and/or treatment of AD. Indeed, APP/PS1 mice treated with daily injection of vitamin D (100 ng/kg BW) for 6 weeks were able to markedly reverse the neuronal loss by decreasing mRNA abundance of apoptosis-promoting genes (caspase-3, Bax) and increasing apoptosis-inhibiting gene Bcl-2. These effects most likely contributed to their improvement in spatial learning and memory as shown by Morris Water Maze and Novel Object Recognition Test [26]. Importantly, the AD-alleviating, neuroprotective effects of vitamin D have been recapitulated in recent clinical trials. In a 12-month randomized, double-blind, placebo-controlled trial, Jia and others were able to demonstrate that compared to the AD group with placebo (starch granule), AD group with vitamin D supplement (800 IU/day) had a significant reduction in plasma Aβ42 and related biomarkers along with improved cognitive function that was tested through a standardized neuropsychological assessments, Wechsler Adult Intelligence Scale-Revised (WAIS-RC) and Mini-Mental State Examination (MMSE) [24]. The same group conducted another 12-month randomized controlled trial with participants diagnosed with mild cognitive impairment (MCI) and reported a significant improvement in the Full Scale Intelligence Quotient (FSIQ) and WAIS-RC scores in the AD group supplemented with vitamin D compared to the control group that received placebo [25]. The ability of vitamin D to restore cognition may be attributed to significantly reduced oxidative stress and increased length of telomere, a DNA sequence at the end of a chromosome whose shortening occurs with age and can predict the rate of MCI or AD progression [185,186]. Interestingly, high levels of vitamin D can cause hypercalcemia and may affect neuronal function. Animal models with high vitamin D intake or increase in the active form of circulating vitamin D by various genetic manipulation have been shown to be associated with reduced brainwave activity, impaired cognitive function, and premature aging [187,188,189]. Hence, precautionary measures should be considered during future clinical trials to assess efficacy and safety of vitamin D supplementation in AD patients. Collectively, the robust findings from both animal and human studies point vitamin D supplementation as a promising and convenient therapeutic approach to mitigate cognitive decline and AD.

## 3. The Role of Minerals in AD Pathogenesis

Minerals are trace elements that are typically found in plants, fruits, meats, and fish. Many of them play an important role in nutrient metabolism or other cellular homeostatic functions by serving as an obligatory cofactor to facilitate enzymatic reactions. Recent studies further indicate antioxidant and anti-inflammatory actions for some of the minerals that may be relevant in brain health and AD development (Table 2).

**Table 2:** Summary on the role of minerals in AD, cognition, amyloid β, and Tau pathology.

Intervention	Minerals	Human Studies	Animal Studies
Dietary intake/ Supplementation/ higher circulating levels	Mg	Lowers risk of cognitive impairment [190,191]	↓ Aβ, ↓ pTau, ↑ cognition [192,193,194,195,196,197]
	Se	↓ Aβ [198], associated with improved cognition [199,200,201] or cognitive dysfunction [202,203]	↓ Aβ, ↓ pTau, ↑ cognition [204,205,206,207,208,209,210,211,212,213]
	Fe	Associated with cognitive impairment [214] or no association with AD risk [215]	↓ Cognition [216], ↓ Aβ, ↓ pTau [217]
	Cu	Association with increased AD risk [218], ↓ cognition [219]	↓ Aβ, ↓ cognition [220,221,222], ↓ Aβ [223]
	Zn	-	-
Deficiency/ restriction/ lower circulating levels	Mg	Association with AD and cognitive impairment [224,225,226,227,228]	-
	Se	Lower levels associated with AD [229,230,231,232,233,234,235]	-
	Fe	-	↓ Aβ, ↓ pTau [236,237,238,239]
	Cu	-	-
	Zn	-	-

↑ = Increase Aβ/pTau or improved cognition, ↓ = decrease Aβ/pTau/dopamine or impaired cognition, Aβ—amyloid beta, pTau—phosphorylated Tau.

### 3.1. Magnesium

Magnesium (Mg) is one of the essential minerals present in a variety of food sources. It stabilizes protein, nucleic acid, lipid membranes, and is a cofactor for several enzymes involved in carbohydrate and lipid metabolism, and is essential for cellular and hormonal signaling [240]. Being crucial for neuronal transmission function [241], Mg is also linked with multiple neurological and neurodegenerative disorders [242,243,244]. Mg blocks the calcium channel in N-methyl-d-aspartate (NMDA) receptors while low Mg may potentiate excitotoxicity, thereby leading to neurotoxicity and neuronal cell death. Hence, Mg deficiency may lead to impaired glutaminergic neuronal activity [245] which is implicated in neurological disorders such as Parkinson’s, epilepsy, and AD [246]. Studies in humans have reported lower systemic Mg levels in AD patients compared to healthy controls [224,225,226,227]. In contrast, few studies have shown no significant changes in blood Mg levels between healthy individuals and AD patients, although these studies were limited by having a small sample size with less power. A recent meta-analysis conducted in 2022 concluded that a significant Mg deficiency exists in subjects diagnosed with MCI or AD [228]. These findings suggest that Mg deficiency may be either the result of low dietary intake of Mg or the consequence of disease progression. Within the brain, low levels of Mg were also observed in the entorhinal cortex, Ammon’s horn, and globus pallidus regions in AD patients [247]. Reduced Mg amount in the AD brain may be attributed to lower circulating Mg levels caused by its reduced dietary intake, or defective Mg transport mechanism. The findings of higher dietary Mg intake are associated with a lower risk of MCI indicating a potential neuroprotective effect of Mg intake or supplementation [190,191]. Supporting these clinical results, 23-month-old rats drinking magnesium-L-threonate-containing water for 24 days significantly improved synaptic function by increasing the number of functional presynaptic release sites, and enhanced learning and working memory as assessed by T-maze and Morris Water Maze [192]. Mg supplementation in AD mice not only reduced tau hyperphosphorylation [193] but also inhibited Aβ-induced neuroinflammation [194,195] by promoting APP cleavage [196], and by increasing clearance of Aβ fibril through modulating BBB permeability [197].

### 3.2. Selenium

Selenium is an essential trace element mainly found in muscle and thyroid gland. It is a vital component of many enzymes (i.e., selenoproteins) that help synthesize and protect DNA, and regulate thyroid function and reproduction [248]. Clinical studies have investigated the link between circulating levels of selenium and cognitive function, amyloid status, or AD risk [229,230,231,232,233,234]. Serum concentrations of selenium and its related total antioxidant status (TAS) [235] or the selenium content in erythrocytes among the AD patients [233] were significantly lower compared to those in healthy elderly group. Similarly, a cross-sectional study on 469 elderly individuals from rural counties in China reported that higher serum levels of selenium are strongly associated with lower serum Aβ42 and Aβ40 [198], indicating a possible neuroprotective role of selenium. Cardoso and colleagues [199] showed in a large cohort study that blood selenium status in older men but not women are positively associated with cognitive performance, suggesting a sex difference in potential positive effects of selenium on brain health. In contrary, others have reported opposite outcomes related to selenium levels and cognitive function. A case–control study by Koseoglu and colleagues [249] measured arsenic and selenium levels in the nails and hair of AD patients and healthy age-matched subjects. The results showed that AD individuals have a higher amount of the two elements in the nails and hair compared to the healthy controls. Higher levels of serum selenium were also positively correlated with cognitive dysfunction in Chinese elderly individuals [202]. It is interesting to note that, in agreement with these findings, Vinceti and others [203] have recently reported a significant inverse relationship between selenium levels in the cerebrospinal fluid (CSF) and hippocampal volume in subjects diagnosed with MCI, thus indicating a potential negative role of selenium in AD development. With that said, intervention studies provide a positive look on selenium. A small randomized, double-blinded, controlled trial comprising patients with AD has shown that co-supplementation of selenium and probiotics improves cognitive function as assessed by MMSE [200]. Consistent with this outcome, a recent meta-analysis of six clinical studies that examined the effects of selenium concluded that the supplementation significantly increases the anti-oxidant glutathione peroxidase activity and enhances cognitive health in either MCI or AD individuals as assessed by MMSE, ADAS-Cog, or Controlled Oral Word Association Test—Verbal fluency (COWAT) [201].

The beneficial role of selenium on neuroprotection and cognition has been largely supported in animal studies [204,205,206,207,208,209,210]. Baldinotti and others [211] reported that in a mouse model of AD induced by intracerebroventricular (ICV) infusion of STZ, intragastric pre-treatment with selenium in the form of octylseleno-xylofuranoside can prevent cognitive and memory decline that may be mediated by decreased lipid peroxidation and its modulatory effects on neurotransmission, as evidenced by changes in acetylcholinesterase and monoamine oxidase. Using a similar STZ-induced AD model in rats, Hashemi-Firouzi and others also demonstrated that oral gavage of selenium nanoparticles (SeNP) or polyvinyl alcohol-coated SeNP for one month significantly enhanced hippocampal BDNF and TAC, lowered malondialdehyde and amyloid plaques, as well as markedly alleviated cognitive and memory deficits as assessed by novel object recognition and passive avoidance learning tests [212]. SeNP was also shown for its ability to enter cells via endocytosis in Aβ-treated PC12 cells to effectively inhibit ROS and inflammatory and apoptotic responses while promoting expressions of BDNF and phosphorylation of AKT and CREB [250]. The underlying mechanisms for the effects of selenium may at least partly be attributed to enhanced AKT signaling since pre-treating PC12 cells with AKT inhibitors completely reversed the anti-inflammatory and neuroprotective effects. This is consistent with a higher enrichment of differentially expressed proteins, in particular those involved in insulin/IGF1-related pathways in the hippocampus and cortex of APP/PS1 mice following treatment with sodium selenate for two months [251], underscoring the importance of restoring insulin signaling in the CNS. In addition, selenium may alleviate AD-related pathology and symptoms through its inhibitory effect on Fe^2+^, Cu^2+^, and Zn^2+^-induced Aβ plaque aggregation [213].

### 3.3. Iron

Iron is a mineral that is widely present in dietary sources and available as a dietary supplement. It is mainly known as an essential element that carries oxygen for hemoglobin synthesis. In the CNS, it plays an important role in neurotransmitter cycling, myelin production, synthesis of ATP and ADP, and enzyme functions. Most of the iron in the brain (90%) is in the form of ferritin, and only 0.05% is present in unstable iron pool. The iron accumulation in the brain is accelerated after the age of 60 [252]. Due to this association with age, brain iron has been the focus of attention in the field of age-related cognition and neurodegenerative diseases such as AD.

The link between circulating levels of iron and AD or cognitive function is mixed. The NHANES data from 2011–2014 reported a negative association between serum iron levels and cognitive impairment in individuals 60 years or older [214]. In another study, serum concentrations of iron were lower in AD patients than those in age-matched control subjects, but no significant correlation was observed between serum iron levels and AD-related genes including APP, PSEN1/2, and APOE4 [253]. Iron concentrations in the erythrocytes of AD patients have also been reported to be inversely related to AD severity measured by CDR scores [229]. On the other hand, Schiepers and colleagues [254] have shown that while high serum ferritin correlated with poor sensorimotor speed and information-processing speed at baseline, the association disappeared over 3 years. Moreover, peripheral iron concentrations in AD patients were found to be unrelated to cognitive scores in a meta-analysis of case–control studies [215]. No genetic overlap was observed between peripheral iron biomarkers such as serum iron, transferrin, and ferritin and AD risk. Conversely, individuals at increased genetic risk of developing AD did not present serum iron elevation.

Measurement of iron accumulation in the brain by MRI has provided more consistent results in AD or other forms of dementia [255,256,257,258,259]. Patients homozygotic for hemochromatosis or iron overload have increased iron deposits in the putamen, hippocampus, and thalamus which is associated with increased dementia incidence and delirium [259]. In their case–control study, Jouini and others [255] demonstrated that AD patients have higher iron concentrations in the cerebrospinal fluid (CSF) compared to cognitively normal subjects, and this was found to be age-dependent. Interestingly, serum iron, ceruloplasmin, and transferrin were lower in those AD subjects. It is possible that defective iron metabolism in the periphery leads to iron transporters like DMT-1 and ferroportin in the blood–brain barrier (BBB) to take up more ferrous iron and release it in the brain resulting in iron overload and neuroinflammation, an established pathological feature of AD [260]. Consistent with this, Spotorno and colleagues [256] observed that iron accumulation in different regions of the cortex in AD, specifically the inferior temporal gyrus, is correlated with Tau accumulation in the same regions. Quantitative susceptibility was shown to mediate the relationship between cortical thickness and Tau deposits, indicating a potential causal role of iron burden in disease progression. However, brain accumulation of iron was not related to age or severity of cognitive deficits in spite of being increased in AD vs. healthy control subjects [257]. The different outcomes may be due to differences in sample size, comparison methods, and the degree of AD progression between the two studies.

Most animal studies support the beneficial effects of reducing iron in AD or age-related cognitive decline. When treated with an iron chelator deferiprone in drinking water for 12 weeks, rabbits with AD-related pathology due to high cholesterol diet feeding were able to significantly lower their systemic iron levels and reduce Aβ42 and Tau phosphorylation in the hippocampus [236]. Similarly, treatment with other iron chelators such as deferasirox and deferoxamine has been shown to be effective in decreasing age-related iron accumulation and suppressing oxidative stress, inflammation, and amyloid deposition in the brain of aged and AD rodents [237,238,239]. The neuroprotective effects of iron chelators could be partly mediated by the shift of microglial pro-inflammatory M1 to anti-inflammatory M2 activation in the brain [238]. The toxic effects of iron overload leading to ferroptosis [261], or iron-induced cell death, appear to be related to AD since the deletion of cortical ferroportin 1—a non-home iron exporter that is downregulated in AD brain—promotes hippocampal atrophy and memory deficits that are reversed following treatment with ferroptosis inhibitors [216]. Others have reported contradictory findings by demonstrating that compared to the vehicle-treated group, mice treated with ferrous sulfate in drinking water for 8 months markedly decreased Aβ42 deposition, Tau phosphorylation, and neuronal apoptosis, which were associated with lower iron levels in the brain [217]. The discrepancy may be attributed to differences in animal models and design, but also to the extent of saturation of systemic and/or brain iron levels [262].

### 3.4. Copper

Copper (Cu) is the third most abundant essential mineral in humans [263], and the brain accommodates the second-highest amount of copper after the liver [264]. It plays a vital role in CNS development, neuromodulation, angiogenesis, and hypoxia management [265], thus its deficiency leads to severe neurological dysfunctions. Excess of cellular copper levels, or copper toxicity, can also result in serious neurotoxicity due to its redox activity and generate reactive oxygen species (ROS). Disruption in copper homeostasis has been associated with neurodegeneration and AD [266]. Copper is widely distributed throughout the brain including the frontal cortex, caudate nucleus, temporal lobe, substantia nigra, striatum, and cerebellum [267]. High concentrations of polyvalent mineral cations (i.e., transition metals) are found to be localized in senile plaques of AD brains [268,269,270,271], and among these, copper can cause more deleterious effects in the pathophysiology of AD [272,273]. In a Chicago community-based prospective study [219], the cognitive function and daily intake including copper and saturated and trans fats were assessed in subjects 65 years or older over a six-year period. The results showed that among people regularly consuming diets high in saturated and trans fats, a faster cognitive decline occurs in those taking a higher copper intake either through Cu-containing supplements or foods. These findings suggest that ingestion of free-floating copper from sources such as drinking water and copper supplements could serve potentially as a causal factor in the onset of AD by inducing greater copper pooling in the brain [218].

A number of animal studies support the deleterious role of copper. Singh and colleagues [220] demonstrated an increased Aβ production and neuroinflammation in APPswe mice following treatment with low Cu (copper sulfate; 0.13 mg/L in drinking water) vs. vehicle for 90 days [220]. Further, high levels of copper may provoke inflammatory responses in the brain which in turn interfere with amyloid plaque degradation. Kitazawa and others demonstrated that mouse monocyte BV2 cells exposed to copper significantly diminished Aβ or LPS-induced phagocytosis while increasing pro-inflammatory cytokines such as IL-1β, IL-6, and TNF-α [221]. Consistent with this, copper exposure to 2-month-old 5xFAD mice in drinking water for 3–12 months led to neuroinflammation and lower expression of outward Aβ-transporting receptor, low-density lipoprotein-related receptor protein-1 (LRP1), compared to 5xFAD control group. Cholesterol-fed rabbits—a model for AD—that are exposed to trace amounts of copper in drinking water were able to promote Aβ accumulation including senile plaque-like structures in the hippocampus and temporal lobe and were severely impaired in their ability to learn a trace conditioning task [222]. Similarly, Wistar rats placed in drinking water containing 2% cholesterol and trace amounts of Cu (3ppm) for two months displayed higher Cu levels in both plasma and the hippocampus, as well as higher Aβ (1–42)/(1–40) ratio in the cortex and hippocampus [274].

One of the major underlying mechanisms for the AD-promoting effects of copper seems to be primarily mediated through its physical interaction with Aβ peptide and the consequent production of ROS. Aβ peptides have two efficient Cu-binding sites, and the high affinity of copper ions to Aβ peptides increases the proportions of α-helix and β-pleated sheet structures of the amyloid aggregates leading to increased formation of senile plaques [275,276,277]. The ability of copper to bind to Aβ peptides that exacerbate cellular toxicity has been shown to be dependent on a pH of 7.0 and higher concentrations of free circulating copper levels. Removing Cu II ions by a specific chelator in rat PC12 cells effectively reduced Aβ42 aggregation and decreased ROS levels, suggesting that through competitive inhibition of Cu II ion binding to amyloid peptides, the severity of AD-related pathology can be minimized [278]. In line with these findings, mouse models with severe neurodegeneration such as 5xFAD and CVN mice exhibit 25% higher copper amount in the cortex compared to the PSAPP mouse model that is known for having very limited neurodegeneration, suggesting that elevation of amyloid-incorporated Cu content is associated with AD severity [279]. It is interesting to note that others have shown a marked reduction of copper content within the brain areas such as the frontal cortex, amygdala, and hippocampus [280,281,282], thus raising a possibility of a causal link between low copper and AD progression. This concept is supported in a study that showed elevated Cu levels in mice with APP deletion, while overexpression of APP was associated with reduced Cu levels in APP23 mice [223]. Treating these AD mice with Cu in drinking water increased its levels in the brains and surprisingly and significantly lowered Aβ peptides. A similar outcome was observed when Cu was added to APP-transfected cells. Differences in transgenic models and experimental designs including sex, the dose of supplemented Cu, and the severity of AD pathology and/or symptoms may have contributed to the conflicting results from these studies.

### 3.5. Zinc

Zinc is an essential trace element in human health and is an integral part of many physiological and biochemical signaling cascades, enzymatic pathways, cell structure, and the immune system. Therefore, disruption of zinc homeostasis can lead to serious neurodegenerative and neurological diseases such as AD and amyotrophic lateral sclerosis (ALS) [283]. A number of studies have established an association between zinc and cognitive health in humans [284,285,286]. Subjects 60 years or older from the NHANES study between 2001 and 2004 showed an inverse relationship between zinc intake and cognitive decline [287]. As part of the Korean Brain Aging Study for Early Diagnosis and Prediction of Alzheimer’s disease (KBASE), a 2017 study observed that in cognitively healthy subjects aged between 55–90, lower serum zinc levels were not related to Tau accumulation or AD-signature cerebral glucose metabolism, but were significantly associated with Aβ deposition in the brain [288]. These results are in line with markedly lower concentrations of zinc and selenium and higher levels of copper/zinc ratio in AD that are associated with cognitive impairment as assessed by MMSE scores [235]. Rivers-Auty and others [289] determined the neuroprotective role of zinc by observing induction of NLRP3-inflammasome complex and accelerated cognitive deficits in APP/PS1 male mice placed on a zinc-deficient diet. Importantly, APP/PS1 mice with NLRP3 deletion were protected from zinc deficiency. These findings are in contrast to the effects of zinc in its interaction with Tau and related neurotoxicity. Li and colleagues [290] have demonstrated that Zn^2+^ is capable of binding the third repeat unit of the microtubule-binding domain of Tau (Tau-R3) and creating a Zn^2+^-Tau-R3 complex which, compared to Tau-R3 alone, can generate higher levels of ROS in Neuro-2A (N2A) cells. In addition, the complex was taken up more readily by the N2A cells and significantly reduced the number and length of these neurons’ axons and dendrites. Apart from its interaction with Tau protein, zinc has been shown to possess a high tendency to bind to Aβ peptides and the ability to form the Zn-Aβ oligomers that have stronger toxicity than Aβ alone by potently inhibiting hippocampal LTP and inducing microglial activation to an overdrive [291,292].

## 4. Conclusions and Future Perspectives

In the last two decades, we witnessed a countless number of human and animal/cell studies that examined the role of nutrients and, in particular, micronutrients whose absolute necessity for cellular functions, nutrient metabolism, and neurophysiology have been validated. While we could not cover all existing literature and rather focused on the most relevant findings for each vitamin and mineral to provide a balanced breadth and depth of what is known at its current state, studies discussed in this review to a large extent support the view that these essential micronutrients, with the exception of iron and copper, might be able to at least help delay the progression of AD through various mechanisms. As somewhat anticipated, many studies suggest that certain vitamins and minerals with high antioxidant and anti-inflammatory properties such as vitamin C, E, B12, and selenium may be able to improve cognitive performance by effectively reducing ROS generation and pro-inflammatory mediators such as NLRP3-inflammasome complex and its downstream signaling in the brain, thereby preserving neuronal health. Other micronutrients such as vitamins A, K, and magnesium that have the capacity to directly affect APP cleavage, Aβ synthesis, and degradation, and transport Aβ peptides or their fibrillar forms via modulating the BBB permeability might help alleviate cognitive decline during disease progression, while vitamins such as niacin (vitamin B3) and pyridoxine (vitamin B6) may contribute to neuroprotection from various insults by maintaining axonal stability, promoting DNA repair, and enhancing neurotransmitter synthesis/release. Moving forward, it would be clinically meaningful to assess the synergistic efficacy of different micronutrients in combination and also determine if any neuroprotective and AD-mitigating effects can be interpreted in a sex-dependent manner. Nonetheless, it is important to note that high consumption of fat-soluble vitamins, e.g., vitamin A, D, and E, have been demonstrated to induce neurotoxicity and requires careful evaluation of the dose of these vitamins before providing them as a supplementation. Other essential fatty acids such as α-linolenic acid and linoleic acid (collectively known as vitamin F) have also shown a potential therapeutic role in AD and cognitive function that deserve further research [293,294]. Furthermore, emerging studies suggest a strong association between gut health and cognitive function [295]. Gut dysbiosis resulting from over-exposure to unhealthy/processed foods and improper nutrient metabolism is thought to alter the gut immune response and barrier permeability that is causally linked to cognitive impairment [295]. Beyond any direct effects of vitamins and minerals on the CNS, identifying and harnessing the role of these important micronutrients in promoting microbial diversity and gastrointestinal (GI) immuno-metabolic responses will provide us with a natural therapeutic strategy to maintain brain health and/or alleviate cognitive deficits. From a therapeutic standpoint, this would give us a valuable opportunity to design and offer differential treatment approaches to AD patients that might be also suffering from GI problems such as inflammatory bowel disease.

## Data Availability

No data were generated for this publication.
